# Net micromineral requirements for maintenance and growth of ewe lambs at the latter fattening period

**DOI:** 10.5713/ajas.19.0390

**Published:** 2019-12-24

**Authors:** Ya Qian Jin, Na Ding, Xiao Gao Diao, Sheng Chen Yu, Jun Xing Zhao, Jian Xin Zhang

**Affiliations:** 1 College of Animal Science and Veterinary Medicine, Shanxi Agricultural University, Taigu, Shanxi 030801, China; 2 College of Animal Science and Technology, Yangzhou University, Yangzhou, Jiangsu, 225009, China; 3 Collaborative Innovation Center for Efficient and Safe Production of Livestock, Shanxi Agricultural University, Taigu, Shanxi 030801, China

**Keywords:** Sheep, Micromineral Requirements, Maintenance, Growth, Comparative Slaughter

## Abstract

**Objective:**

The objective of this study was to investigate the net micromineral (Cu, Fe, Mn, Zn) requirements for maintenance and growth of Dorper×Jinzhong crossbred ewe lambs at their latter fattening period.

**Methods:**

Thirty 1/2 Dorper × 1/2 Jinzhong crossed F1 ewe lambs (35±0.5 kg of body weight [BW]) were used and divided into five groups in a randomized design for a comparative slaughter trial. At the beginning of the experiment, six lambs were randomly selected and slaughtered at 35 kg BW to determine their initial body composition. When their BWs reached 43 kg, another six lambs fed *ad libitum* were slaughtered to serve as an intermediate slaughter group. The retained eighteen lambs were randomly distributed into three groups and offered one type of feed at 100%, 65%, and 40% of the *ad libitum* intake. When the lambs fed *ad libitum* reached a BW of 50 kg, the three groups were slaughtered. The body composition (muscle, fat, bone, blood with viscera, skin, and wool) were weighted, ground, mixed, and subsampled for mineral content analysis.

**Results:**

The net maintenance requirements of Cu, Fe, Mn, and Zn were 0.017, 0.160, 0.004, and 0.067 mg/kg BW/d, respectively, and the net growth requirements per 100 grams of average daily gain ranged from 0.48 to 0.51 mg of Cu, 2.63 to 2.17 mg of Fe, 0.12 to 0.15 mg of Mn, and 2.07 to 2.00 mg of Zn, respectively, for Dorper × Jinzhong crossed ewes from 35 to 50 kg BW.

**Conclusion:**

Our results suggest that the micromineral requirements for both maintenance and growth of Dorper × Jinzhong crossbred ewe lambs were quite different from the recommendations of NRC (2007), except for Zn.

## INTRODUCTION

Minerals are separated into macrominerals and microminerals according to the amounts required by animals [1]. They are inorganic elements that are essential for the body’s metabolic processes, such as activating enzymatically catalyzed reactions, serving as components of certain transport proteins and hormones, or maintaining water and electrolyte balance. Besides, minerals are involved in the acid-base status, osmotic pressure, and cellular permeability [2], which play vital roles in the prevention of diseases and disorders. Either excess or deficiency in one element may result in metabolic disorders, and interactions among these minerals in the body could interfere in their availability [3], which might impair animal’s production, reproduction, immunity and even survival. Thus, the estimation of mineral requirements should be a priority in small ruminant production systems to reduce the costs of production and the excretion of inorganic elements in the environment without adverse effects on animal performance [4].

The Jinzhong sheep is a native breed in Shanxi province, China with large physique, strong adaptability, and high disease resistance [5]. Dorper breed is specialized for meat production and is characterized by hardiness, early sexually maturity, rapid growth and excellent meat quality [6]. To improve growth performance and carcass quality, Dorper sheep have recently been introduced in Shanxi to crossbreed with Jinzhong sheep, and Dorper × Jinzhong crossbreed has become the dominant crossbreed for lamb production in Shanxi [7]. Nevertheless, the diets formulated for sheep in China are based on information recommended by international committees on animal nutrition. A recent study of Dorper × Jinzhong crossed ram lambs suggested that energy requirements are lower than the requirements recommended by the National Research Council (NRC) and Agricultural and Food Research Council (AFRC) [7], and therefore, it is expected that mineral requirements will also vary from AFRC recommendations.

Net mineral requirements for maintenance of ruminants can be estimated by various methods including endogenous losses, comparative slaughter, and radioisotopes [8]. Taking into consideration of accuracy, environmental safety and strong operability [9,10], comparative slaughter has recently been accepted as a better method to estimate mineral requirements for sheep and goats [8,11–15]. Previous studies have mostly addressed the trace element requirements of lambs during their initial stages of growth [12,15], however, such reference data are sub-optimal for lambs’ diet formulations in their latter fattening periods, as the mineral requirements vary with the gender, breed, final body weight (BW), and developing stages [1,16]. Therefore, this work estimates the net requirements of micro minerals (Cu, Fe, Mn, Zn) for the maintenance and growth of Dorper × Jinzhong crossbred female lambs at the latter fattening period.

## MATERIALS AND METHODS

The experiment was conducted at the Experimental Station of Shanxi Agricultural University, Shanxi, China. During the experimental period, the temperature ranged from 16°C to 28°C, with timely ventilation. All animal procedures were approved by the Animal Care and Use Committee of Shanxi Agricultural University, and were in compliance with the university’s guidelines for animal research (Protocol number: SXAU-[2014]-8).

### Animals and management

Thirty Dorper × Jinzhong crossed ewe lambs (35±0.5 kg) were randomly selected from the Sheep Farm of the Ecological Farming Cooperatives in Taigu, Shanxi Province, and confined in individual stalls (3.0 m×0.8 m) equipped with feeders and automatic water suppliers. Prior to the experiment, all lambs were injected with ivermectin at a dosage of 0.2 mg/kg of BW to eliminate parasites and fed an experimental diet *ad libitum* for a 10 days adaptation period. At the beginning of the experiment, six lambs were selected randomly and slaughtered as the reference group (RG) to determine their initial body composition. The remaining 24 lambs were equally assigned to four groups in a randomized design according to the level of dry matter intake (DMI) (*ad libitum* 1, *ad libitum* 2, 65% of the *ad libitum* intake and 40% of the *ad libitum* intake). Lambs in *ad libitum* groups, 65% of the *ad libitum* group and 40% of the *ad libitum* group were expected to achieve BW gains of approximately 300, 150, and 0 g/d, respectively, according to NRC [1]. When the lambs fed *ad libitum* reached 43 kg BW, one of *ad libitum* groups were slaughtered to serve as an intermediate slaughter group (I-AL). The other three groups were slaughtered finally (F-AL, F-65%, F-40%) when lambs fed *ad libitum* reached a BW of 50 kg. The duration of the experiment was 68 days.

The experimental diet was formulated as a pelleted mixed diet with a concentrate to roughage ratio of 45 to 55 (dry matter [DM] basis), and the ingredients and nutrient contents are shown in Table 1. Animals were fed twice daily at 8 am and 6 pm and had free access to clean water. The orts of the previous day in the *ad libitum* group were collected and weighted each day in the morning for feed intake calculation. The amount of feed offered to the *ad libitum* group was adjusted each day based on the feed intake of the previous day to ensure a 10% residue. The amount of feed offered to the two restricted groups was also adjusted based on the feed intake of the *ad libitum* group from the previous day. Individual samples of feed supplied, and orts were collected daily and pooled for further analysis. All lambs were weighed every 10 days.

### Sampling and measure

Slaughtering and sampling procedures were performed as previously reported [5] with small modifications. Prior to slaughter, the lambs were weighted (recorded as BW) and then fasted for 16 h, their shrunk BW was measured immediately after the fast. After stunning via CO_2_ inhalation, the lambs were slaughtered by exsanguination, and the weights of the blood, carcass, head, feet, hide, skin (with wool), viscera, and adipose tissue (removed from internal organs) were recorded. The digestive tract, including the esophagus, rumen, reticulum, omasum, abomasum, and small and large intestine, were weighed before and after the removal of the contents. The carcass and head were split at dorsal midline into two identical halves; the right half of the carcass, head, and anterior and posterior feet were dissected into muscle, bone, and fat. The bone was fully ground with a bone grinder; the muscle, fat, viscera with blood, and skin (without wool on) were cut separately into small pieces, and fully ground with a mincing machine. After grinding, a 500-g sample of each part was collected and stored at −20°C for further analysis. Wool samples were separated from skin and taken. Finally, the body was divided into six components including muscle, fat, bone, blood + viscera, skin, and wool.

The feed and orts were dried at 105°C at least 8 h for DM analysis using the procedure described by Association of Official Analytical Chemists (AOAC [17]). A sample (100 g) of each body component except the wool was lyophilized for 72 h. Ashes of all samples were obtained via complete combustion in a muffle furnace at 600°C for 6 h. Subsequently, the ash was solubilized in HCl [14], and the concentrations of Fe, Mn, Cu, and Zn were measured by the atomic absorption spectrophotometric method [18].

### Data calculation

#### Maintenance

For intermediate and final slaughter groups, the initial body mineral elements were calculated through regression equation developed between body mineral elements content and empty body weight (EBW) of baseline animals. The initial EBWs of intermediate and final slaughter groups were calculated according the regression equation between EBW and BW. Retained mineral element was calculated as difference between the final and initial body mineral element content. Body mineral content was calculated the sum of mineral content value of the six body components. The net requirement for maintenance (mg/kg of EBW) was estimated using the negative intercept of the linear regression of daily trace elements retained (mg/kg of EBW) on trace elements intake (mg/kg of EBW), which represented unavoidable trace element losses of lambs [19].

#### Growth

Allometric equations for the relationship between the mineral contents (mg) of the empty body and the EBW (kg) were developed based on the data of lambs fed *ad libitum* to predict the body mineral composition [20,21]. The derivatives of these equations were taken to calculate the composition of the gain at various EBWs. The net mineral requirement for growth (i.e., retained mineral) was then calculated as the difference between the mineral content of the empty at different intervals of EBW. The net mineral requirements for gain in BW were obtained via the linear regression equation of EBW on BW.

### Statistical analysis

The statistical analysis was performed using SAS 9.0 (SAS Institute. Inc., Cary, NC, USA). The BW, average daily gain (ADG), average dry matter intake (ADMI), EBW, mineral intake, mineral retained, body composition, and body mineral content were analyzed using PROC analysis of variance; the Duncan test was used for the post hoc analyses. Linear regressions were performed using PROC REG. Observations with a studentized residual >2.5 or <−2.5 were considered to be outliers. The assumptions of the models, in terms of homoscedasticity, independency, and normality of the errors, were examined by plotting residuals against predicted values; p<0.05 was considered to be statistically significant in all data.

## RESULTS

### Maintenance requirements

Equations for the initial body mineral contents estimation are shown in Table 2. The retained minerals were calculated as a difference between the final and initial body mineral contents. The results of mineral intake and retained, and the growth performance of lambs at different feeding levels are given in Table 3. The initial BWs of all lambs were the same (p>0.05), whereas the final BWs and ADG are influenced positively by intake levels (p<0.05), providing higher feed intake and therefore greater final BWs and ADG. In accord with ADMI, mineral intake of Cu, Fe, Mn, and Zn were markedly decreased as the intake level decreased (p< 0.05). For Cu and Mn, the mineral retention was decreased linearly in response to the decreasing intake levels (p<0.05). However, the Fe retention was greater in ewes fed *ad libitum* than those fed at 40% feed (p<0.05) but did not differ from animals fed at 65% intake. The retention of Zn in the *ad libitum* group was higher compared with the other two groups, but there was no significant difference between 65% and 40% restricted feed intake groups.

The relationship between minerals retention and minerals intake is shown in Table 4. The intercept of the regression was the trace element requirements for maintenance. The maintenance requirements of Cu, Fe, Mn, Zn were 0.017, 0.160, 0.004, and 0.067 mg/kg BW/d, respectively, for Dorper × Jinzhong crossed ewes from 35 to 50 kg BW.

### Growth requirements

The RG, I-AL, and F-AL groups were used in estimating the mineral requirements for growth. Ewe lambs with restricted feed intake were not included in the prediction because their growth pattern differed from those fed *ad libitum*. The growth performance, weights of tissues (including muscle, fat, bone, blood with viscera, skin and wool), and mineral contents of EBW in the three groups are shown in Table 5. The final body weight and EBW of lambs in the three slaughter groups were linearly increased as the experiment time prolonging, while the ADG and ADMI had no difference between I-AL and F-AL groups. Weights of muscle and fat in the three groups exhibited the same pattern of changes (F-AL>I-AL>RG, p< 0.05); Bone and blood with viscera weights were greater for lambs in F-AL group than those in the RG but did not differ from lambs in I-AL group; For skin and wool, the weights in F-AL group were significantly greater compared with in the other groups, but little difference was found between I-AL and F-AL groups. Body mineral contents of Cu, Fe, Mn, Zn linearly increased with increasing EBW (p<0.05).

As shown in Table 6, the logarithmic allometric equations including the EBW and body mineral quantities, which were significant (p<0.001) and provided a good data fit (R^2^ between 0.81 and 0.97), were constructed to predict the Cu, Fe, Mn, Zn contents in the lambs. Deriving the logarithm regression equations from Table 6, the equations to estimate the mineral composition of the empty weight gain in ewes weighting from 35 to 50 kg were obtained as shown in Table 7.

Based on equations in Table 6, the body micromineral components at different BWs (35 to 50 kg) were calculated (Table 8). With an increase in BW, the absolute contents of all mineral (Cu, Fe, Mn, Zn) increased, whereas the relative contents of mineral to EBW responded differently with Cu and Mn increasing, but Fe and Zn decreasing. The net mineral requirements for growth with varying ADGs (100 to 250 g) at varying BWs (35 to 50 kg) are listed in Table 9.

## DISCUSSION

In this study, we found that final BW, ADG, and EBW of lambs receiving different feed intakes increased as intake levels increased, which was similar with the results for Dorper × Hu crossed lambs and Caninde goats reported by Zhang et al [12] and Souza et al [11], respectively.

To correctly estimate the mineral requirements of growing sheep, we should have an exact knowledge of mineral deposition in the body, which is effected by gender, body weight and feeding intensity. In the present study, the retention of the trace elements (Cu, Fe, Mn, Zn) in empty body have decreases of approximately 82.76%, 129.37%, 66.67%, and 61.17%, respectively, as feed intake decreased, which agrees with a previous report [11]. The mineral intake, which was calculated based on the recorded feed intake of lambs, had a trend in accordance with ADMI. Moreover, the ratios of mineral retention to mineral intake of minerals (Cu, Fe, Mn, Zn) were decreased from 10.03% to 3.40%, 0.88% to 0.51%, 0.12% to 0.08%, and 4.25% to 3.25%, respectively, as feed intake increased. Similar results were discovered in data of Zhang et al [12]. For the reason, there is a higher proportion of unavoidable loss in feed-restricted groups, which would lead to a lower efficiency of mineral deposition.

In current study, the ADG of lambs fed with diets formulated according to NRC [1] in both I-AL and F-AL groups were lower than expected (300 g/d), which suggested NRC likely underestimates the nutritional requirements of Dorper × Jinzhong crossed ewes. In addition, there were only minor differences in the ADG of ewes in different BW ranges (35 to 43 kg for I-AL group, and 35 to 50 kg for F-AL group), this differed from the results of Dorper × Jinzhong crossed rams that showed the ADG of lambs during early growing stage were greater than the whole growth period [22]. The ADG in current study were lower than results of Dorper × Hu crossed rams reported by Zhang et al [12], which showed the growing rate of males was greater than females. In accordance with the feeding plan, the final BW and EBW of lambs increased as ages increased, which may lead to the differences of muscle, fat, bone, blood, skin and wool weight among the three slaughter groups. However, there is a consistent pattern for growth in young animals, that was first for bone, second for muscle, and last for fat [22]. As the slaughter weight of our lambs increased, the EBW increased about 44%, which was greater than the increase of muscle and bone (33.26% and 17.60%, respectively), but much less than fat (213.79%). Thus, we deduce that the weight gain of lambs at the latter fattening period is mainly from the growth of adipose tissue, in accordance with Gomes et al [23].

As expected, the amounts of minerals examined (Cu, Fe, Mn, Zn) increased significantly with higher BWs, similar results were also found for the German Merino Landsheep [24]. The deposition of Cu and Mn during the fattening period increased, respectively from 4.47 to 4.94 mg/kg EBW and from 0.83 to 1.02 mg/kg EBW. However, a decrease in the concentration of Fe and Zn was observed (from 56.19 to 47.53 mg/kg EBW and from 24.99 to 24.17 mg/kg EBW).

The mineral requirements of growing sheep are directly linked to the mineral deposition in the body, which varies with genotype, gender, developmental stage, and experimental condition. The ewes used in this study were at the latter finishing stage, wherein adipose tissues grow more quickly in relation to general body development, which is also rapid in adults in comparison to the young animals. To date, few studies have estimated the nutritional mineral requirements of lambs in the later stage of growth. Thus, a comparison of existing work in the literature is difficult.

In the NRC [1], the mineral requirements of copper, iron, manganese and zinc were estimated with the factorial method for sheep. In addition, the estimation of mineral requirements for maintenance in recommendations, such as ARC [19], NRC [1], were mostly based on the fecal endogenous losses, and the mineral retention is determined from the difference between the mineral intake and the total excretions. However, it does not necessarily reflect the total mineral required for maintenance, because, in addition to feces and urine, there are other inevitable losses of minerals, for example, through the skin, sweat, and so on [20]. Therefore, the comparative slaughter method, a direct method to determine the retained mineral content, was used in present study to estimate the mineral requirement of Dorper × Jinzhong crossed ewe lambs.

As a very important element, copper (Cu) plays key roles in the actions of many crucial enzyme systems [1]. The deficiency is manifested as anemia, enzootic ataxia and osteochondrosis, but excessive copper intake would cause haemolytic, liver tissue damage, even death [1]. Because the availability of Cu is reduced in the presence of molybdenum, sulfur and iron, the net requirement of Cu is difficult to determine [2]. In current study, the net requirement of Cu for maintenance was 0.017 mg/kg BW/d, which was greater than those reported by the NRC [1], which estimated the net Cu requirement for maintenance as 0.004 mg/kg BW/d for the postweaning lambs kept in feedlots. For Dorper × Jinzhong crossed ram lambs, the maintenance requirement of Cu was 0.013 mg/kg BW/d from 35 to 50 kg [21], slightly lower than those for ewe lambs in present study. Animals in our study were in the later finishing period, whose requirements of Cu for maintenance were lower than those reported by Zhang et al [12], which have reported the net maintenance requirements of Cu for Dorper × Hu crossed females as 0.0238 mg/kg BW/d from 20 to 35 kg BW. Expressed as mg/d, the maintenance requirements of Cu estimated in our study was (0.595 to 0.850) mg/d, which was lower than the data reported by Ji [25] as 1.12 mg/d for ewes from 20 to 35 kg BW. These results indicated that maintenance requirement of Cu was reduced for lambs with growth. The net requirements for growth of Cu increased from 0.48 to 0.51 mg/d per 100 grams live weight gain for ewes from 35 to 50 kg. Little difference was found among animals with the same ADG but different BW, maybe this is the reason why the growth requirement recommendations of NRC [1] are invariable per gram live weight gain for postweaning lambs. In present study, the growth requirement for lambs of 35 kg with ADG 100 g was 0.48 mg/d, which was much less than the values (1.02 mg/d) estimated by Jin et al [21], but greater than the results (0.106 and 0.17 mg/d) reported by NRC [1] and Zhang et al [12], respectively.

Iron (Fe) is the most abundant trance element of the body and mainly found in hemoglobin which is involved in transporting oxygen and carbon dioxide to and from the tissues and the lung [1]. Besides, iron can activate enzymes, such as succinate dehydrogenase, hydroxylases, peroxidases, to participate in the metabolism. Iron supplies are rarely inadequate for older farm livestock, in which the major problem is presented by excess dietary Fe [26]. Net requirement of Fe for maintenance was 0.160 mg/kg BW/d, or (5.6 to 8.0) mg/d from 35 to 50 kg in present study, which was close to values (0.131 mg/kg BW/d, and 5.98 mg/d) reported by Zhang et al [12] for Dorper × Hu crossed ewe lambs from 20 to 35 kg BW, and by Ji [25] for Dorper × thin-tailed Han crossbred ewes from 20 to 35 kg BW. Compared with recommendation in NRC [1] for lambs weaned, the value in our study was nearly ten times greater (0.014 mg/kg BW/d). However, the result in this study was approximately 30% lower than the value (0.228 mg/kg BW/d) estimated for rams of the same breed and body weight, which manifested that genders had a great influence on the Fe requirement for maintenance. The growth requirements of Fe decreased from 2.63 to 2.17 mg/d per 100 grams live weight gain for ewes from 35 to 50 kg. For lambs of 35 kg with ADG 100 g, the net requirements for growth of Fe (2.63 mg/d) was less than the value (5.5 mg/d) recommended by NRC [1], and lower than the growth requirement (6.28 mg/d) for Dorper × Jinzhong crossed rams, but greater than the result (1.76 mg/d) reported by Zhang et al [12].

Manganese (Mn) concentration in the body is low, but it is an essential element for ruminants. Manganese deficiency leads to bone abnormalities, interrupted reproductive activity, ataxia in newborns and defective lipid and carbohydrate metabolism. The requirements of Mn for sheep were estimated by the factorial method in NRC [1], and the endogenous loss (2 μg/kg BW) was used as maintenance requirement. However, the maintenance requirement of Mn estimated by the comparative slaughter method in present study was 0.004 mg/kg BW/d, which was greater than the recommendation reported by NRC [1] and the value (0.0027 mg/kg BW/d) estimated by Zhang et al [12], but lower than the result (0.006 mg/kg BW/d) reported by Jin et al [21]. Expressed as mg/d, the maintenance requirements of Mn estimated in our study was (0.14 to 0.20) mg/d, which was similar to the data reported by Ji [25] as 0.22 mg/d for ewes from 20 to 35 kg BW. Thus, the maintenance requirement of Mn is greatly influenced by the research methods. The growth requirements of Mn increased from 0.12 to 0.15 mg/d per 100 grams live weight gain for ewes from 35 to 50 kg. For lambs of 35 kg with ADG 100 g, the net requirements for growth of Mn (0.12 mg/d) was lower than the growth requirement (0.22 mg/d) for Dorper × Jinzhong crossed rams, but greater than the values (0.047 and 0.025 mg/d) reported by NRC [1] and Zhang et al [12], respectively.

Zinc (Zn) plays a major role in growth and health of all animals and is involved in numerous metalloenzymes, gene expression and appetite. Zn deficiency is a worldwide nutritional problem, the incidence rate of which is ≥25% of the world’s population estimated conservatively [27,28]. Lambs having insufficient Zn in the diet may have inhibited development of the spleen and thymus and exhibit symptoms such as anorexia, lose wool and have swollen hocks. Therefore, it is necessary to estimate Zn requirement accurately to prevent Zn disorders. It was considered that daily endogenous loss of Zn was 0.076 mg/kg BW, which was accepted as maintenance requirement of Zn by NRC [1]. In the current study, the Zn requirement for maintenance was 0.067 mg/kg BW/d, which represented 2.35 to 3.35 mg/d when the ewes grew from 35 to 50 kg BW, which was slightly lower than both the recommendation of NRC [1] and value (0.0816 mg/kg BW) estimated by Zhang et al [12], but greater than the result (2.34 mg/d) reported by Ji [25]. In addition, the estimated value in present study was very close to our previous finding in ram lambs of 0.069 mg/kg BW [21]. These results suggested that gender has little impact on the maintenance requirement of Zn, which agrees with previous observations [25]. The growth requirements of Zn decreased from 2.07 to 2.00 mg/d per 100 grams live weight gain for ewes from 35 to 50 kg. For lambs of 35 kg with ADG 100 g, the net requirement for growth of Zn (2.07 mg/d) was close to the values in previous reporters [1,12,21].

The net maintenance requirements of Cu, Fe, Mn, and Zn were 0.017, 0.160, 0.004, and 0.067 mg/kg BW/d, respectively, for Dorper × Jinzhong crossed ewes from 35 to 50 kg BW. The net growth requirements per 100 grams of ADG ranged from 0.48 to 0.51 mg of Cu, 2.63 to 2.17 mg of Fe, 0.12 to 0.15 mg of Mn, and 2.07 to 2.00 mg of Zn as lambs grew from 35 to 50 kg. The requirements for both maintenance and growth of elements were quite different from the recommendations of NRC [1], except for Zn. In addition, gender wasn’t considered in the NRC [1] for growing lambs, while our results indicated that gender may have a marked influence on the requirement of Cu, Mn, and Fe. Thus, it is necessary to estimate the requirement for both male and female lambs to provide accurate reference data for sheep production. Because of great difference between the results of Zhang et al [12] and Ji [25], we can’t get an accurate conclusion about the trend of requirements with lambs’ growth.

## Figures and Tables

**Table 1:** Ingredients and nutritional composition of the mixed diet

Dietary ingredient	g/kg	Nutrient composition	
Corn stalk	250	DM (g/kg)	885.5
Sunflower seed null	95	ME (MJ/kg)	82.8
Alfalfa hay	100	CP (g/kg)	97.6
Corn grain	350	NDF (g/kg)	387.2
Soybean meal	70	Cu (mg/kg)	13.12
Rapeseed meal	40	Fe (mg/kg)	310.26
DDGS	40	Mn (mg/kg)	100.3
Wheat bran	40	Zn (mg/kg)	149.84
Dicalcium phosphate	5		
Salt	5		
Mineral/vitamin premix1)	5		

DDGS, distillers dried grains with solubles; DM, dry matter; ME, metabolizable energy; CP, crude protein; NDF, neutral detergent fibre; Cu, copper; Fe, iron; Mn, manganese; Zn, zinc.

1) The premix provides the following per kg of diet: Vit A 15,000 IU; Vit D 5,000 IU; Vit E 50 mg; Fe 90 mg; Cu 12.5 mg; Mn 50 mg; Zn 100 mg; Se 0.3 mg; I 0.8 mg; Co 0.5 mg.

**Table 2:** Equations for the initial body mineral contents estimation of lambs

Items	Equation	R^2^	RMSE	p-value
EBW	EBW (kg) = (−0.963±6.301)+(0.837±0.178)×BW (kg)	0.876	0.48	0.004
Cu	Lg Cu (mg) = (0.446±1.086)+(1.129±0.744)×lgEBW (kg)	0.635	0.02	0.026
Fe	Lg Fe (mg) = (1.196±0.709)+(1.372±0.485)×lgEBW (kg)	0.700	0.01	0.011
Mn	Lg Mn (mg) = (0.040±0.434)+(0.913±0.298)×lgEBW (kg)	0.677	0.01	0.035
Zn	Lg Zn (mg) = (0.619±0.422)+(1.532±0.290)×lgEBW (kg)	0.871	0.01	0.013

RMSE, root mean square error; EBW, empty body weight; BW, body weight; Cu, copper; Fe, iron; Mn, manganese; Zn, zinc; Lg or lg means log_10_.

**Table 3:** The growth performance, mineral intake and retained minerals of lambs as influenced by different feeding levels

Items	Level of intake			SEM	p-value

	F-AL	F-65%	F-40%		
NO. of lambs	6	6	6	-	-
Initial BW (kg)	35.02^a^	34.83^a^	35.07^a^	0.16	0.82
Final BW (kg)	49.73^a^	42.85^b^	35.72^c^	1.42	<0.0001
ADG (g/d)	216.42^a^	117.89^b^	−5.15^c^	22.73	<0.0001
ADMI (g)	1,915.75^a^	1,239.76^b^	708.43^c^	119.99	<0.0001
EBW (kg)	40.82^a^	34.79^b^	29.72^c^	1.14	<0.0001
Mineral intake (mg/kg EBW/d)					
Cu	0.289^a^	0.220^b^	0.147^c^	0.014	<0.0001
Fe	16.269^a^	12.359^b^	8.274^c^	0.815	<0.0001
Mn	4.873^a^	3.702^b^	2.478^c^	0.244	<0.0001
Zn	2.423^a^	1.841^b^	1.232^c^	0.121	<0.0001
Mineral retained (mg/kg EBW/d)					
Cu	0.029^a^	0.011^b^	0.005^c^	0.002	<0.0001
Fe	0.143^a^	0.103^a^	−0.042^b^	0.023	<0.0001
Mn	0.006^a^	0.004^b^	0.002^c^	0.000	<0.0001
Zn	0.103^a^	0.061^b^	0.040^b^	0.008	<0.0001

SEM, standard error of the mean; BW, body weight; ADG, average daily gain; ADMI, average dry matter intake; EBW, empty body weight; Cu, copper; Fe, iron; Mn, manganese; Zn, zinc.

F-AL, final slaughter group that were fed *ad libitum*; F-65%, final slaughter group that were fed at 65% level of *ad libitum* intake; F-40%, final slaughter group that were fed at 40% level of *ad libitum* intake.

a–c Values followed with different superscript letters on the same row represent a significant difference (p<0.05).

**Table 4:** Equations for to predict the net micromineral requirement for maintenance of lambs

Items	Equation	R^2^	RMSE	p-value	Net requirements	

					mg/(kg EBW/d)	mg/(kg BW/d)
Cu	RCu = (−0.021±0.003)+(0.160±0.012)×ICu	0.911	0.004	<0.0001	0.021	0.017
Fe	RFe = (−0.200±0.031)+(0.022±0.002)×IFe	0.835	0.041	<0.0001	0.200	0.160
Mn	RMn = (−0.005±0.001)+(0.003±0.0002)×IMn	0.880	0.001	<0.0001	0.005	0.004
Zn	RZn = (−0.082±0.016)+(0.080±0.007)×IZn	0.879	0.017	<0.0001	0.082	0.067

RMSE, root mean square error; EBW, empty body weight; RCu represents retention of Cu, ICu represents intake of Cu, the same as Fe, Mn, Zn; Cu, copper; Fe, iron; Mn, manganese, Zn, zinc.

**Table 5:** Performance and body micromineral contents of lambs throught different growth periods

Items	Slaughter groups1)			SEM	p-value

	RG	I-AL	F-AL		
NO. of lambs	6	6	6	-	-
Days on feed (d)	-	38	68	-	-
Initial BW (kg)	35.73^a^	34.55^a^	35.02^a^	0.30	0.22
Final BW (kg)	35.73^c^	42.81^b^	49.73^a^	1.42	<0.0001
ADG (g/d)	0.00^b^	217.32^a^	216.42^a^	28.69	<0.0001
ADMI (g)	0.00^b^	2,024.21^a^	1,915.75^a^	263.06	<0.0001
EBW(kg)	28.34^c^	36.22^b^	40.82^a^	1.34	<0.0001
Muscle (kg)	10.86^c^	13.07^b^	14.48^a^	0.42	<0.0001
Fat (kg)	3.52^c^	7.98^b^	11.06^a^	0.80	<0.0001
Bone (kg)	4.16^b^	4.54^a^	4.89^a^	0.09	0.003
Blood (kg)	4.89^b^	5.53^a^	5.67^a^	0.12	0.005
Skin (kg)	2.54^b^	2.73^b^	3.10^a^	0.07	0.006
Wool (kg)	0.52^b^	0.65^b^	1.10^a^	0.07	<0.0001
Body Cu (mg)	126.52^c^	164.52^b^	201.61^a^	7.73	<0.0001
Body Fe (mg)	1,591.22^c^	1,770.97^b^	1,938.70^a^	38.64	<0.0001
Body Mn (mg)	23.63^c^	36.94^b^	40.80^a^	1.97	<0.0001
Body Zn (mg)	708.38^c^	879.18^b^	986.05^a^	30.05	<0.0001

SEM, standard error of the mean; BW, body weight; ADG, average daily gain; ADMI, average dry matter intake; EBW, empty body weight; Cu, copper; Fe, iron; Mn, manganese, Zn, zinc.

1) RG, reference group; I-AL, intermediate slaughter group that were fed *ad libitum*; F-AL, final slaughter group that were fed *ad libitum*.

a–c Values followed with different superscript letters on the same row represent a significant difference (p<0.05).

**Table 6:** The logarithmic allometric equations to predict the Cu, Fe, Mn, Zn contents in the lambs

Items	Equation	R^2^	RMSE	p-value
EBW	EBW (kg) = (−3.978±2.403)+(0.918±0.058)×BW (kg)	0.948	1.1867	<0.0001
Cu	Lg Cu (mg) = (0.402±0.158)+(1.170±0.103)×lgEBW (kg)	0.901	0.0261	<0.0001
Fe	Lg Fe (mg) = (2.458±0.098)+(0.511±0.064)×lgEBW (kg)	0.817	0.0161	<0.0001
Mn	Lg Mn (mg) = (−0.882±0.167)+(1.559±0.110)×lgEBW (kg)	0.935	0.0276	<0.0001
Zn	Lg Zn (mg) = (1.533±0.068)+(0.906±0.045)×lgEBW (kg)	0.967	0.0113	<0.0001

RMSE, root mean square error; EBW, empty body weight; BW, body weight; Cu, copper; Fe, iron; Mn, manganese; Zn, zinc; Lg or lg means log_10_.

**Table 7:** Equations to estimate the mineral composition of the empty weight gain in lambs

Items	BW (kg)				Equation

	35	40	45	50	
EBW (kg)	28.15	32.74	37.33	41.92	
Cu (mg/kg EBW)	5.21	5.34	5.46	5.57	Cu = 2.9525×EBW^0.170^
Fe (mg/kg EBW)	28.68	26.64	24.98	23.61	Fe = 146.6969×EBW^−0.489^
Mn (mg/kg EBW)	1.32	1.44	1.55	1.65	Mn = 0.2046×EBW^0.559^
Zn (mg/kg EBW)	22.59	22.27	22.00	21.76	Zn = 30.9121×EBW^−0.094^

BW, body weight; EBW, empty body weight; Cu, copper; Fe, iron; Mn, manganese; Zn, zinc.

**Table 8:** The body micromineral components at different body weights of lambs

Items	BW (kg)			

	35.00	40.00	45.00	50.00
EBW	28.15	32.74	37.33	41.92
Cu (mg)	125.29	149.51	174.32	199.64
Fe (mg)	1,580.15	1,706.94	1,825.30	1,936.72
Mn (mg)	23.87	30.20	37.06	44.40
Zn (mg)	701.87	804.80	906.37	1006.78
Cu (mg/kg EBW)	4.45	4.57	4.67	4.76
Fe (mg/kg EBW)	56.13	52.13	48.89	46.20
Mn (mg/kg EBW)	0.85	0.92	0.99	1.06
Zn (mg/kg EBW)	24.93	24.58	24.28	24.02

BW, body weight; EBW, empty body weight; Cu, copper; Fe, iron; Mn, manganese; Zn, zinc.

**Table 9:** The net micromineral requirements for growth with varying average daily gains at different body weights of lambs (mg)

Items	ADG	BW(kg)			

		35.00	40.00	45.00	50.00
Cu	100	0.48	0.49	0.50	0.51
	150	0.72	0.74	0.75	0.77
	200	0.96	0.98	1.00	1.02
	250	1.20	1.23	1.25	1.28
Fe	100	2.63	2.44	2.29	2.17
	150	3.94	3.66	3.44	3.25
	200	5.26	4.88	4.58	4.33
	250	6.57	6.10	5.73	5.41
Mn	100	0.12	0.13	0.14	0.15
	150	0.18	0.20	0.21	0.23
	200	0.24	0.26	0.28	0.30
	250	0.30	0.33	0.36	0.38
Zn	100	2.07	2.04	2.02	2.00
	150	3.11	3.07	3.03	3.00
	200	4.15	4.09	4.04	3.99
	250	5.18	5.11	5.05	4.99

BW, body weight; ADG, average daily gain; Cu, copper; Fe, iron; Mn, manganese; Zn, zinc.
